# Hydrogen and the Light-Induced Bias Instability Mechanism in Amorphous Oxide Semiconductors

**DOI:** 10.1038/s41598-017-17290-5

**Published:** 2017-12-04

**Authors:** Hongfei Li, Yuzheng Guo, John Robertson

**Affiliations:** 1 Engineering Department, Cambridge University, Cambridge, CB2 1PZ UK; 2 College of Engineering, Swansea University, Swansea, UK

## Abstract

Hydrogen is known to be present as an impurity in amorphous oxide semiconductors at the 0.1% level. Using amorphous ZnO as a simplified model system, we show that the hydrogens pair up at oxygen vacancies in the amorphous network, where they form metal-H-metal bridge bonds. These bonds are shown to create filled defect gap states lying just above the valence band edge and they are shown to give a consistent mechanism to explain the negative bias illumination stress instability found in oxide semiconductors like In-Ga-Zn-O (IGZO).

## Introduction

Amorphous oxide semiconductors (AOS) such as In,Ga,Zn oxide (IGZO) are now widely used as thin film transistors (TFTs) in high resolution liquid crystal displays and as driver transistors of organic light emitting diode (OLED) displays, because of their higher electron mobility than hydrogenated amorphous silicon (a-Si:H)^1–5^. However, there remains the problem that the TFTs suffer from the negative bias light induced stress instability (NBIS), a shift of the gate threshold voltage during negative bias stress and illumination^6–11^. In NBIS, a slightly sub-bandgap light excites electrons from some filled defect states just above the valence band edge to the conduction band, where they produce a persistent photoconductivity (PPC)^7,11^. The defect states responsible are seen in hard X-ray photo-emission^12^, but their cause is still contentious.

There have been various models to account for this instability, involving oxygen vacancies^11,13–16^, oxygen interstitials^17–20^, or hydrogen impurities^18,21,22^, but each of these models has its flaws. The instability has often been attributed to the oxygen vacancy as this is a common defect in oxides and the instability is worse in O-deficient films^15,16^. The O vacancy also has the ‘negative-U’ property^23,24^ which is a mechanism that creates an energy barrier to carrier recombination and thus it explains the persistence of the photoconductivity^11^. However, the problem is that the vacancy states lie too high in energy in the band gap^24,25^ compared their energy as seen by photoemission^12^.

The second model based on oxygen interstitials does produce states in the required energy range^17,18^, as seen in random network models^19^, and these can also have the negative-U like property^18^. However, the problem is that they are an oxygen excess defect while annealing the film in an oxygen excess is known to reduce the instability^15,16^. A related model to the O vacancy model links the instability with the presence of In^1+^ valence sites whose In 6s lone pair states lie in the required energy range. However, this valence is not seen in the photoemission core level spectra^20^.

A final possibility is that it is due to hydrogen. It has been proposed that single hydrogen atoms could undergo a deep to shallow transition and become donor-like^21,22^. However, this defect does not correspond to an oxygen deficiency and there is little other evidence for this mechanism.

Thus, in this paper we propose a new mechanism in which the instability is associated with a complex of two hydrogen atoms at an oxygen vacancy, a V_O_/2H complex. This is an adaptation of the model of Du and Biswas^26^ for hidden hydrogen in c-ZnO. We will show that this defect complex in the amorphous phase gives a consistent explanation of the bias stress instability and its persistent photo-conductivity. The complex gives rise to gap states lying just above the valence band maximum (VBM) as seen by photoemission, it clearly has an oxygen deficiency, it has the negative U property, and it has a low formation energy in the amorphous phase. In its neutral configuration, the hydrogens form 3-center bridging sites, whose vibrational signature has recently been seen experimentally in infra-red (IR) by Bang *et al*.^27^. It should be noted that a considerable hydrogen content of over 10^20^ atoms cm^−3^ is seen in a-IGZO by thermal desorption spectroscopy or secondary ion mass spectrometry (SIMS) ^28–30^, which is many orders of magnitude more than that seen in crystalline (c-) ZnO. This concentration is similar to the density of sub-gap defect states seen in the amorphous films by photoemission^12^, and much larger than the photo-excited carrier density created by NBIS^8^.

Hydrogen has a complicated role in oxide semiconductors. In c-ZnO or a-IGZO, interstitial hydrogen acts as a shallow donor^31,32^. In this case the hydrogen is bonded to an oxygen atom as a O-H bond. However, in the amorphous case, all the 10^20^cm^−3^ hydrogens cannot be acting as donors, most must be compensated in some way. One way would be if the hydrogens were compensated by interstitial O acceptors. However this would not have the necessary O deficiency. We show that the lowest energy of H pairs in a-ZnO is the V_O_/2H state which is self-compensated for E_F_ above 2 eV. In this state, the hydrogens exist as anionic H^-^, forming bridge bonds to metal atoms.

To show this, we study the incorporation of H_2_ and water into an a-ZnO network. We use a-ZnO as a simplifying model of the a-IGZO network^32^, as it has a higher symmetry crystalline phase, c-ZnO. We first create a random network of a-ZnO by density functional molecular dynamics using the CASTEP code^33,34^, and then substitute an H_2_ molecule for an oxygen site and study its different charge states. We then introduce an H_2_O molecule into the *defect-free* network and study its behavior. Finally we consider the migration of the hydrogens from the dissociated water molecule to the oxygen vacancy. These results show that hydrogen or water can passivate the vacancy, but only at the expense of introducing states which lead to the possibility of the NBIS effect.

## Results and Discussion

**Figure 1:**
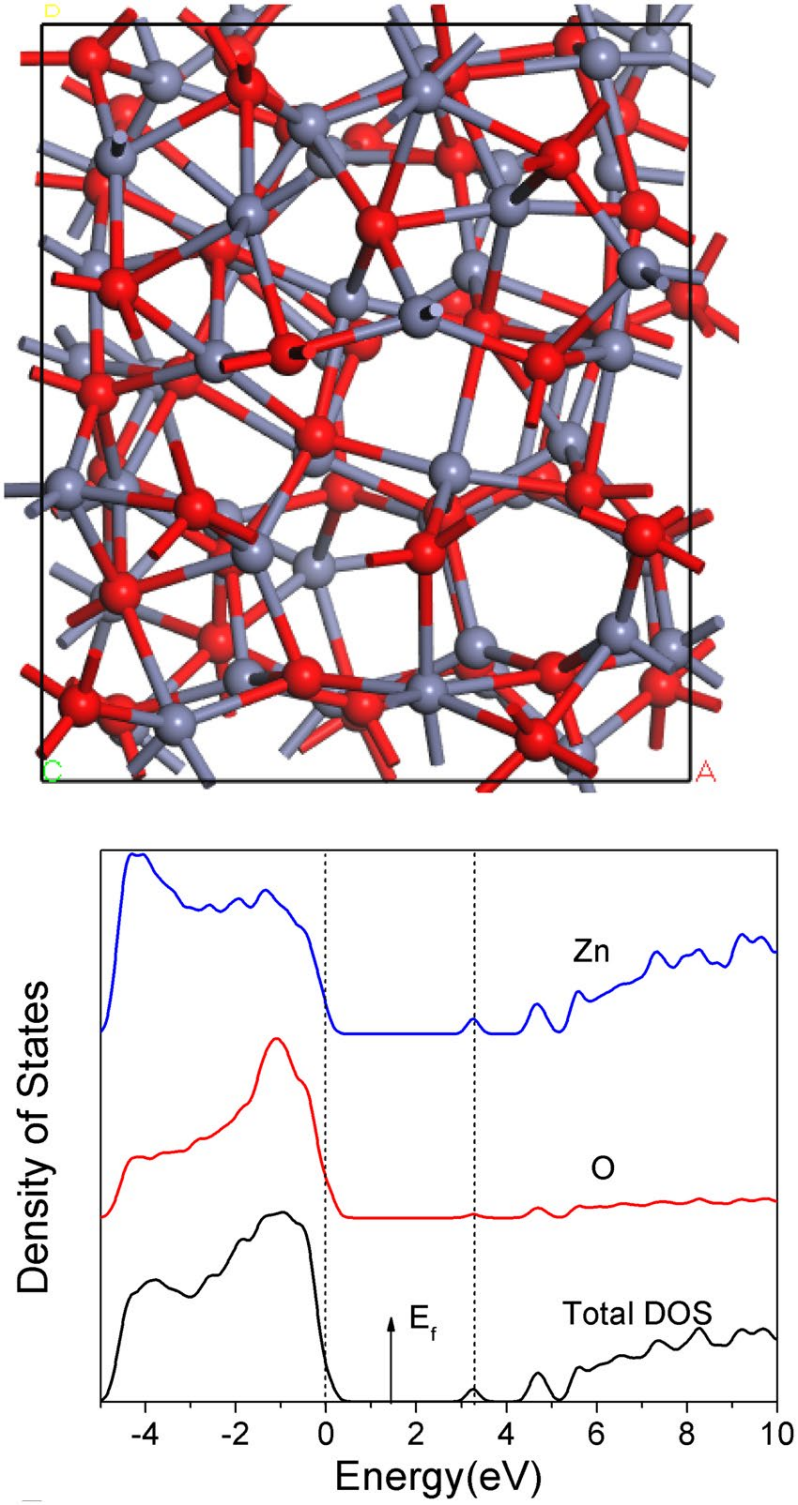
(**a**) A random network of amorphous ZnO. Red balls = O, grey balls = Zn. (**b**) Partial density of states of the amorphous ZnO network of (**a**).

Figure 1 shows the resulting 96 atom random network of ZnO. Most Zn and O atoms are 4-fold coordinated but some are 3-fold coordinated, reminiscent of the undercoordinated defects in a-GaAs^35,36^. Figure 1(b) shows the density of states (DOS) calculated using the screened exchange (sX) hybrid functional^34^. sX corrects the band gap error of simple density functional theory. We see that the band gap is 3.2 eV, slightly smaller than that of the crystal.

**Figure 2:**
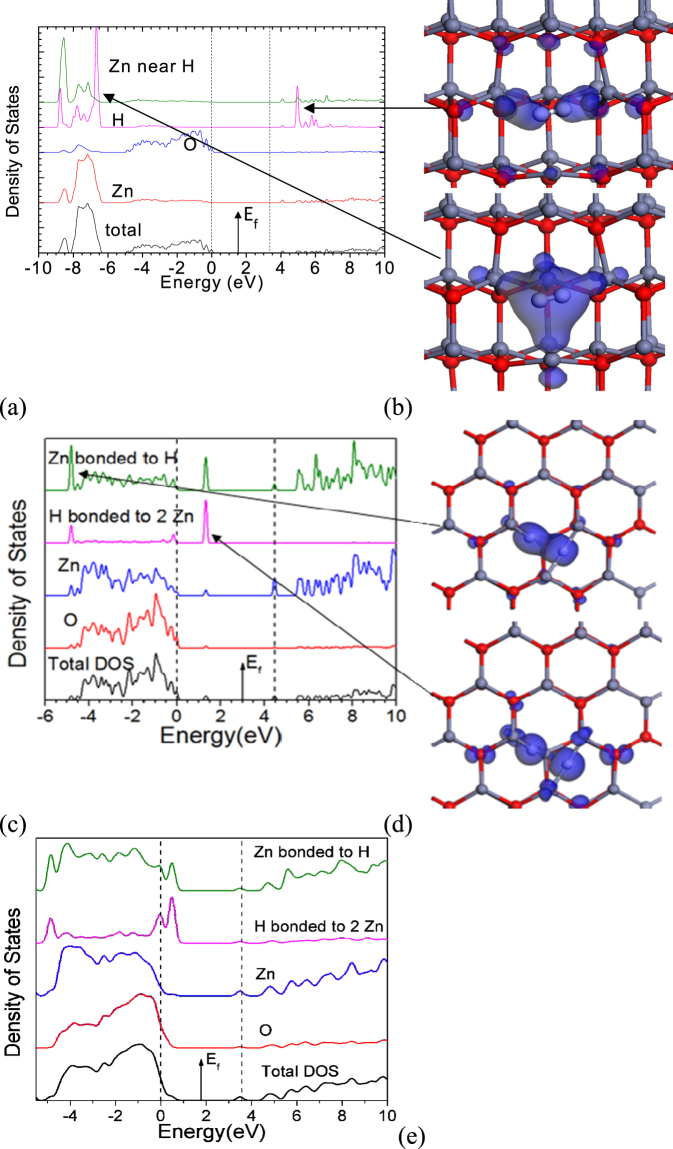
(**a**) Partial density of states on the H site and on the adjacent Zn site, of the V_O_^2+^(2H) complex in crystalline ZnO with an internal H_2_ molecule. (**b**) orbitals of the H-H bonding state at −6.5 eV (bottom) and the H-H antibonding state at +5.5 eV (top). (**c**) Partial density of states of the neutral V_O_^0^(2H) complex in c-ZnO, (**d**) orbitals of the bonding state at −5.5 eV (top) and the gap state at 1.0 eV (bottom). This latter state is the cause of NBIS. The antibonding state around +5.5 eV is unclear in the PDOS. (e) PDOS of the V_O_^0^(2H) complex in amorphous ZnO, equivalent to (**c**). Red balls = O, grey balls = Zn. White balls = H.

We first consider the case of inserting H_2_ into c-ZnO. Figure 2(a,b) shows an H_2_ molecule at an O vacancy in ZnO. Its simplest configuration is the V_O_^2+^(2H) state. Here, the four metal dangling bond orbitals pointing into the vacancy are empty. This leaves two electrons on the two hydrogen atoms. These form a neutral H_2_ molecule trapped inside the vacancy. This molecule gives rise to a filled H-H bonding state lying at −6.5 eV below the ZnO valence band, whose wavefunction is shown in Fig. 2(b), and an empty anti-bonding state lying near + 5.5 eV in its conduction band, as is seen in the partial density of states plots of Fig. 2(a). We find that a H_2_ molecule at a vacancy has essentially the same total energy as it has in free space^26^.

The second configuration of interest is the neutral V_O_°(2H) state, as seen in Fig. 2(c,d). In this charge state, the complex has a completely different configuration of C_2v_ symmetry consisting of two 3-center Zn-H-Zn bridges. Each bridge uses two of the four Zn dangling bond orbitals at the vacancy. Each bridge creates three states; a filled 3-center bonding state lying at −5.5 eV whose wavefunction is shown in Fig. 2(d, upper panel), a filled 3-center state lying at +1.2 eV above the VBM, which is anti-bonding along the Zn-H bonds, whose wavefunction is shown in Fig. 2(d, lower panel), and finally an empty Zn-Zn antibonding state lying well into the conduction band at roughly 5 eV. The energies of the states agree with those found by Du and Biswas^26^. We also note that the PDOS of the neutral V_O_/2H complex in a-ZnO has a very similar energy spectrum to that in c-ZnO, comparing Fig. 2(c,e), showing that the bonding is similar in the two phases, although the gap state does lie slightly closer towards the valence band in a-ZnO.

**Figure 3:**
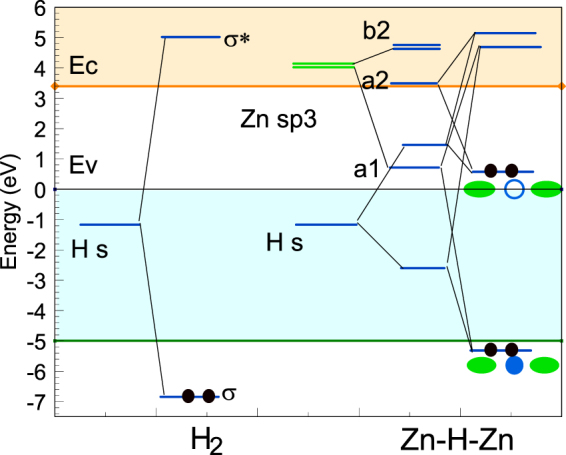
Approximate molecular orbital (MO) diagram of the V_O_/2H defect in ZnO, in the 2+ state (left) and neutral state (right), showing the origin of the local orbitals.

A molecular orbital diagram showing the layout of these states is shown in Fig. 3. The ideal O vacancy has T_d_ symmetry, and its vacancy orbitals form one A_1_ and three T_2_ states. When it acquires C_2v_ symmetry the T_2_ states split into one of A_2_ symmetry and a pair of B_2_ symmetry. Meanwhile, the hydrogen orbitals form a symmetric and antisymmetric pair. The symmetric state interacts with the A_1_ vacancy state and the antisymmetric one interacts with the A_2_ vacancy state, each forming bonding and antibonding configurations. The four available electrons then fill the resulting two bonding states. The A_1_ state lies at −5.5 eV and the A_2_ state, being the key gap state, lies at +1.2 eV. The fact that both of these states are filled means that the H orbitals are overall 100% filled, giving the hydrogens an anionic H^-^ configuration^24^. As the hydrogens have an H^-^ configuration, the H^-^ pair is essentially iso-electronic with the O^2-^ ion.

**Figure 4:**
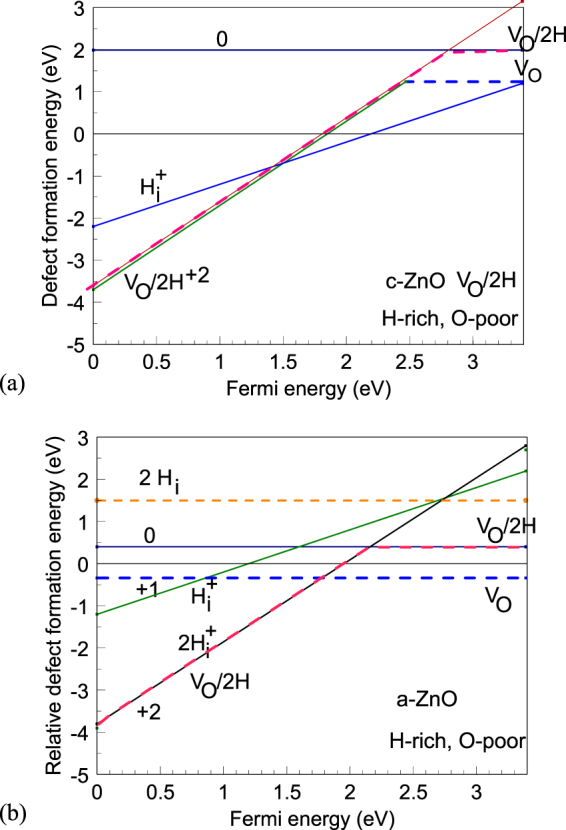
(**a**) Defect formation energy vs. Fermi energy for the V_O_/2H complex for the O-poor, H-rich condition in crystalline ZnO, (**b**) defect formation energy for V_O_/2H complex and interstitial hydrogen in amorphous ZnO for O-poor condition.

Figure 4(a) shows the defect formation energies for the hydrogen interstitial donor and the V_O_/2H complex in c-ZnO. As noted by Du and Biswas^26^, the donor is the more stable configuration over the important range of Fermi energies (above midgap).

Although it is the same V_O_/2H defect complex as in c-ZnO, it has rather different properties in the amorphous phase a-ZnO because of changes in the free energies. This arises because a-ZnO has a lower mass density of 4.62 g/cm^3^ compared to 5.44 g/cm^3^ for c-ZnO. The heat of formation of a-ZnO is calculated to be −2.98 eV per O in sX, compared to −3.44 eV per O in sX for c-ZnO, so the energy of disorder of 0.46 eV is quite large. It should be noted that for the ZnO:H system, the O-poor limit is no longer set by the metal/metal oxide equilibrium but by the H_2_/H_2_O equilibrium to an O chemical potential of μ(O) = −3.34 eV.

The average defect formation energy of the neutral O vacancy is +3.0 eV for a-ZnO in the O-rich condition (μ(O) = 0 eV), compared to +4.80 eV for c-ZnO, a sizable change. This is due to the lower density of a-ZnO which allows the atoms around the vacancy to relax more. +3.0 eV for O-rich corresponds to −0.34 eV for the O-poor condition.

Figure 4(b) shows the calculated defect formation energies of the V_O_/2H complex in a-ZnO for each charge state. We see that the 0 and +2 states are the most stable over the critical range of Fermi energies, above midgap. The H^+^ interstitial is less stable than the 2H/V_O_ complex, over the range of Fermi energies, as seen in Fig. 4(b). Thus, the complex is a negative U defect. This is different to the situation in c-ZnO. This explains the compensation of hydrogen in a-ZnO, and is significantly different to c-ZnO.

The large positive defect formation energy of neutral V_O_/2H in c-ZnO means that the concentration of the V_O_/2H complex or ‘hidden hydrogen’ in c-ZnO is quite low, ~10^16^ cm^−3^. Its much lower formation energy of this defect in a-ZnO means that there is an orders of magnitude higher H concentration in the amorphous phase, even for a lower formation temperature. In addition, the formation energy of the V_O_/2H complex in the H-rich condition in a-ZnO is lower than that for the interstitial H donor configuration H_i_ in Fig. 4(b), so that the compensated V_O_/2H complex configuration is dominant.

Nomura *et al*.^28^ noted that IGZO typically contains a large concentration of hydrogen due to the poor vacuum pumping in the typical sputtering systems used for oxide deposition. Bang *et al*.^27^ identified the infra-red vibrational modes of the Zn-H-metal bridge groups in IGZO, and that they were more thermally stable than the vibrational modes of the O-H group. The greater stability of the Zn-H-metal bridge groups is because these defects are trapped at an O vacancy^37^. In contrast, the hydrogens at O-H bonds are interstitial, and migrate easily, as noted by Wardle *et al*.^38^.

Our calculations show that the states of metal-H bridge bonds are the typical cause of filled gap states at 1.0 eV above the valence band edge seen by X-ray photoemission^12^. Electrons in these states can be excited by photons of energy 2.5 eV into the conduction band and give the observed persistent photoconductivity^6,8,9^. These electrons can drift to the gate dielectric interface^7,9^, giving a charge that causes a negative threshold voltage shift. It should be noted that the V_O_^0^(2H)/V_0_^2+^(2H) group also possesses a negative U, that is its 1+ state is less stable that the neutral or 2+ states. This energy barrier acts, as in the simple O vacancy^23,24^, as a recombination barrier, so maintaining the persistent photoconductivity.

The V_O_^0^(2H)/V_O_^2+^(2H) model has many similarities to the simpler O vacancy model, except that the origin of the photo-excited states is different. In the vacancy model, vacancy states are excited, which actually lie near midgap, whereas in the new V_O_^0^(2H)/V_O_^2+^(2H) case, hydrogen bridge states are excited. These lie just above the valence band maximum and more consistent with photo-excitation by 2.5 eV photons. Thus, the present model is closer to the experimental situation.

**Figure 5:**
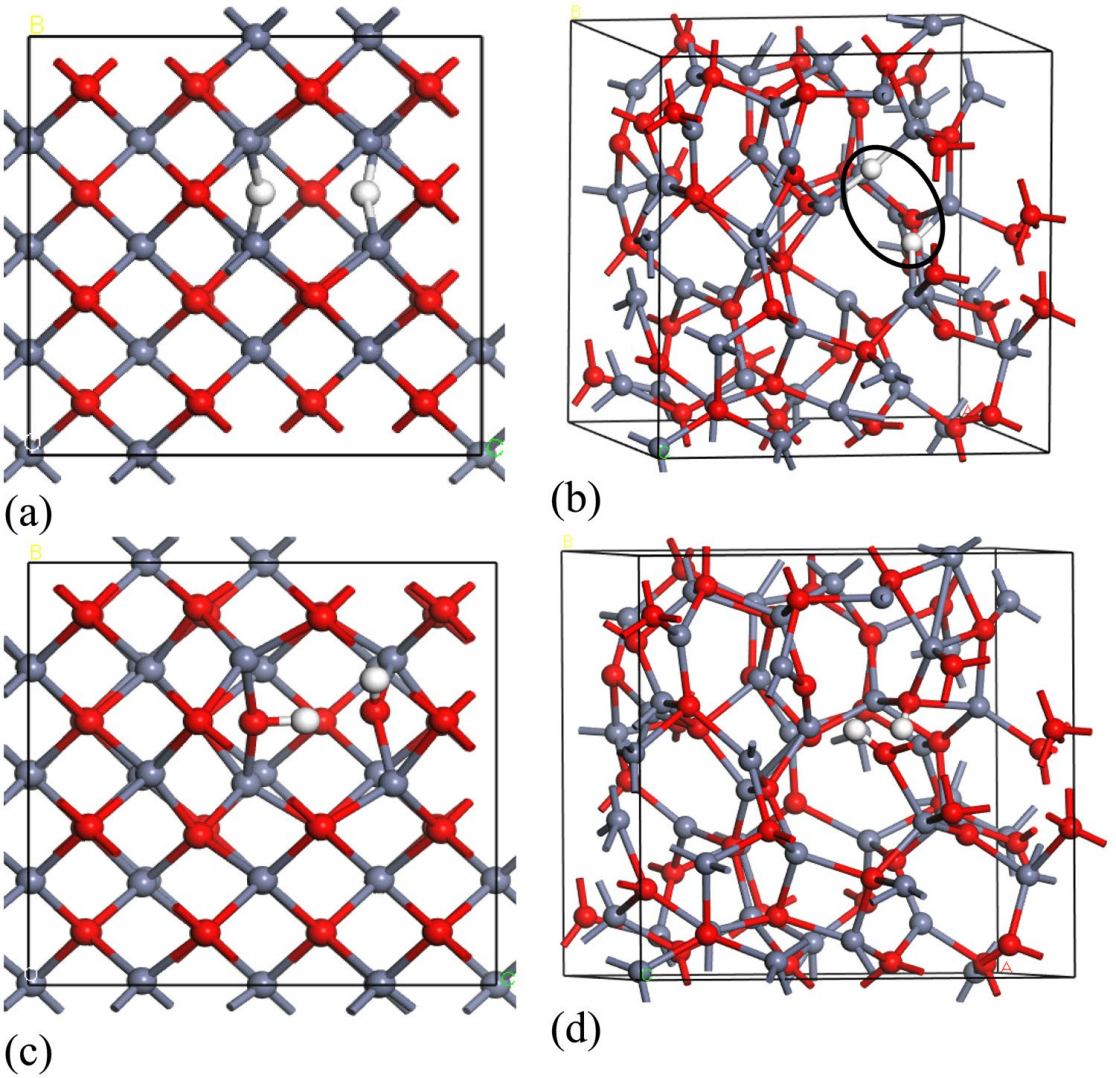
The configurations of the Zn-H-Zn bridges from the V_O_^0^/2H complex in (**a**) crystalline ZnO and (**b**) amorphous ZnO, showing the conservation of local bonding (2H units circled). The configurations of a dissociated water molecule in (**c**) crystalline and (**d**) amorphous ZnO. The hydrogens are bonded covalently to one oxygen and weakly to a second oxygen. Red balls = O, grey balls = Zn. White balls = H.

Overall, the details of the model have been calculated for the binary oxide a-ZnO, but a similar situation applies to other oxides like a-IGZO, ZnSnO_x_ and In_2_O_3_ etc, which all have similar band gaps. ZnO has a 4-fold covalent random network, while the other oxides have more ionic bonding associated with the In or Sn sites. Nevertheless the behaviour of H at an O vacancy is similar in each case, Fig. 5(a,b), in that it gives rise to Zn-H-Zn bridges with filled states in the lower band gap region, which can be photo-excited.

We finally consider the addition of a water molecule to the ZnO network. Figure 5(a,b) shows the water in crystalline and amorphous ZnO. The H_2_O species dissociates into H and OH groups which insert into a Zn-O bond. The OH group attaches itself to the metal dangling bond and the H attaches to the O dangling bond, resulting in two adjacent –OH groups. The OH groups remain close to each other, bound by a weak hydrogen bonding. In effect, each H is bonded strongly to one O site and weakly bonded to the second O site in an asymmetric bridge configuration resembling that the hydrogen sites in water.

**Figure 6:**
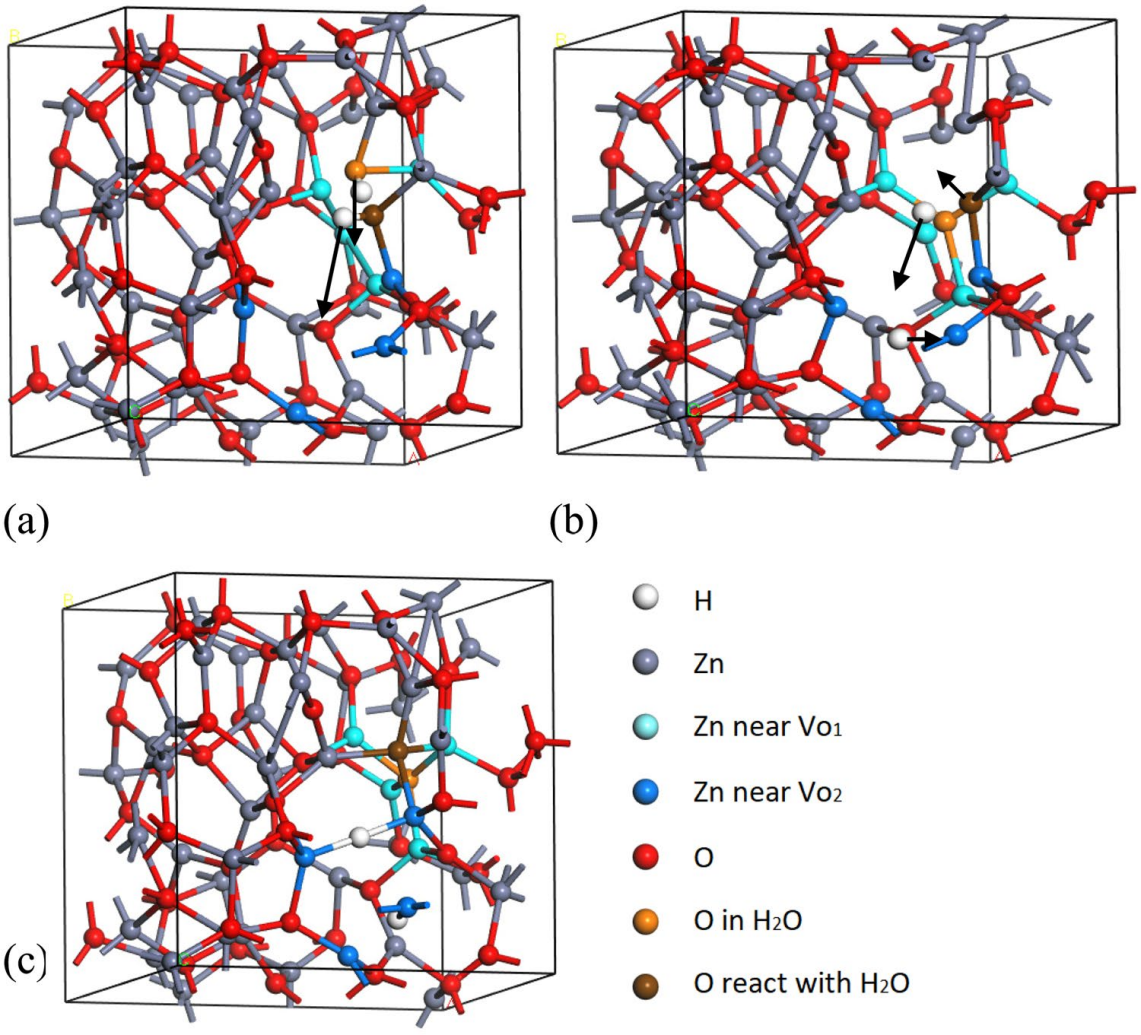
(**a**) A dissociated water molecule in amorphous ZnO, adjacent to two oxygen vacancies. (**b**) the transition state where two hydrogens are moving to one of the vacancies (motion arrowed), and the oxygen is moving to the other vacancy. (**c**) Final state in which the water’s oxygen has reached one of the vacancies, and the two hydrogens have reached the other oxygen vacancy, thereby passivating the two vacancies. Blue and turquoise balls are the Zn atoms adjacent to each of the O vacancies for guidance. White balls = H, as noted in the color key.

An H_2_O group in ZnO consists of an excess of a H_2_ and an O compared to the pure state. Figure 6(a) shows the (ZnO)_n_.1 H_2_O configuration plus two nearby neutral O vacancies. Figure 6(b,c) shows how two hydrogens from the H_2_O group could move to one of the vacancies and the O of the H_2_O could move to the other vacancy, via the transition state of Fig. 6(b). In the final state of Fig. 6(c), the O has filled one of the O vacancies, and thus passivated it. The two hydrogens have filled the other vacancy, and bonded to all the Zn dangling bonds. They have passivated it in the sense that they have removed the deep mid-gap state associated with a neutral O vacancy in ZnO. They have replaced its mid-gap state with states close to the VBM associated with the Zn-H-Zn bridges. The process has removed states in the upper half of the gap and mid-gap traps that affect the operation of the oxide as an n-type transistor. Thus the water molecule has passivated states affecting the transistor action, as seen experimentally^37,38^.

Finally it is interesting to compare the behavior of hydrogen in a-ZnO with that a-Si:H. Although hydrogen passivates the single Si dangling bond in a-Si:H^39^, it seems that hydrogen occurs mostly in pairs in Si or a-Si:H^40–42^. For example in crystalline Si, H will form the H_2_* defect, which is a low energy hydrogen configuration caused by inserting two hydrogens into a Si-Si bond^40^. This two hydrogen defect has an even lower energy in the a-Si:H network, and is also strongly involved in the network growth process of a-Si:H^42^. It is the main defect in the instability mechanisms of a-Si:H^43,44^. In the same way, the two-hydrogen defect is more stable in a-ZnO than in c-ZnO and this causes its leading role in the amorphous oxide semiconductors.

## Summary

In summary, it is proposed that the sizable hydrogen content seen in amorphous oxide semiconductors exist as hydrogen atom pairs trapped at oxygen vacancies. These form metal-H-metal bridge bonds in oxide semiconductors and are the source of the negative bias illumination stress instability observed in these materials. Our calculations show that such bonds give rise to defect states in the lower gap region which are the cause of this instability.

## Methods

The calculations are carried out using 96 atom supercells of amorphous ZnO. These are created by a molecular dynamics (MD) anneal at 2000K for 20 ps, followed by a quench to 300 K in 20 ps. The calculations are carried out using the CASTEP plane wave pseudopotential code^43,44^. It uses norm-conserving pseudopotentials with a cutoff energy of 760 eV. The energies are converged to 10^−5^ eV per atom, and forces to under 0.01 eV/Å. Density functional theory (DFT) greatly under-estimates the band gap of ZnO which can lead to errors, particularly in the description of the donor states of hydrogen. For static calculations, these can be rectified by using hybrid functionals such as the screened exchange functional for the electronic exchange-correlation functional^44–46^. However, a less expensive method is desirable for the MD stage. For this, we use the GGA + U method. This includes an on-site potential U of 5 eV on the Zn 3d states to lower their binding energy^24,47^. However, this is not enough, and we also add a U potential of 5 eV on the O 2p states to further lower the energy of the valence band maximum^48^.

The transition states of intrinsic defects are calculated using the Lany and Zunger^49^ scheme. The formation energy of each charge state is given by

$${{\rm{H}}}_{{\rm{q}}}({{\rm{E}}}_{{\rm{F}}},\mu )=[{{\rm{E}}}_{{\rm{q}}}-{{\rm{E}}}_{{\rm{H}}}]+{\rm{q}}({{\rm{E}}}_{{\rm{v}}}+{{\rm{\Delta }}{\rm{E}}}_{{\rm{F}}})+{{\rm{\Sigma }}}_{\alpha }{{\rm{n}}}_{\alpha }(\mu \alpha +{{\rm{\Delta }}\mu }_{\alpha })$$

where q is the charge on the system, E_q_ is the energy of charged system with a defect, E_H_ is the energy of charged defect-free system. E_V_ is the valence band maximum (VBM) and E_F_ is the Fermi level with the respect to VBM. n_α_ is the number of atoms of species α, μ_α_ is the relative chemical potential of element α. We note that the first two terms are equal to the difference between the total energy of charged defect system and total energy of neutral defect-free system.

The density of c-ZnO is 5.44 g/ cm^3^, the density of a-ZnO is 4.62 g/ cm^3^. The supercell of a-ZnO contains 96 atoms, and has a lattice constant of 11.3 Ǻ. The O vacancy is created by removing one O atom from the supercell, and taking it externally to form half an O_2_ molecule. The network or supercell is then relaxed by GGA. For a-ZnO, several oxygen atom sites are sampled to obtain an average defect formation energy. We take the average of the 6 lowest formation energies from 20 sampled sites (high formation energy sites will have a low probability.)

The water molecule is inserted into the bulk c-ZnO or a-ZnO network at a Zn-O bond. The structure is relaxed at constant volume.
